# Nationwide trends in cigarette, heated tobacco product, e‐cigarette, and multiple‐product use in Japan 2017–2025

**DOI:** 10.1002/pcn5.70409

**Published:** 2026-09-04

**Authors:** Satomi Mizuno, Satoshi Inoura, Toshihiko Matsumoto, Takuya Shimane

**Affiliations:** ^1^ Department of Drug Dependence Research, National Institute of Mental Health National Center of Neurology and Psychiatry Kodaira Japan; ^2^ Department of Nursing, Faculty of Nursing Niigata Seiryo University Niigata Japan

**Keywords:** addiction, nicotine dependence, public policy

## Abstract

**Aim:**

To describe nationally representative trends in overall and product‐specific tobacco use, including cigarettes, heated tobacco products (HTPs), and electronic cigarettes (e‐cigarettes), in Japan from 2017 to 2025.

**Methods:**

Data came from repeated cross‐sectional nationwide surveys of the Japanese population using stratified two‐stage random sampling. Self‐administered questionnaires assessed tobacco use. Past‐30‐day prevalence of overall, product‐specific, and multiple‐product use was estimated using survey‐weighted logistic regression, with marginal predicted prevalence standardized to the 2025 age–sex structure.

**Results:**

Among 16,499 participants, overall past‐30‐day tobacco use declined from 24.3% (95% confidence interval [CI]: 22.6–26.0%) in 2017 to 21.0% (CI: 19.4–22.7%) in 2025. Cigarette use decreased from 20.7% (CI: 19.0–22.3%) to 12.3% (CI: 11.1–13.6%), whereas HTP use increased from 5.3% (CI: 4.4–6.1%) to 11.7% (CI: 10.3–13.0%). e‐cigarette use remained low, ranging from 0.7% to 1.3%. The most common multiple‐product pattern was concurrent cigarette and HTP use, increasing from 2.5% (CI: 1.9–3.1%) to 4.1% (CI: 3.3–4.9%).

**Conclusion:**

Overall tobacco use in Japan declined modestly during 2017–2025, alongside a marked decrease in cigarette use and an increase in HTP use, while e‐cigarette use remained limited. These findings suggest a shift in tobacco use, with increased HTP use partially offsetting reductions in cigarette smoking.

## INTRODUCTION

New nicotine products, such as heated tobacco products (HTPs) and electronic cigarettes (e‐cigarettes), have become increasingly prevalent worldwide alongside conventional cigarettes^1^, ^2^, ^3^, ^4^, ^5^. HTPs heat processed tobacco material or tobacco sticks to generate a nicotine‐containing aerosol, whereas e‐cigarettes heat a liquid that does not contain tobacco leaf and may or may not contain nicotine. Because HTPs and nicotine‐containing e‐cigarettes can efficiently deliver nicotine, concerns have been raised regarding their potential to initiate, maintain, or reinforce nicotine dependence^1^, ^2^, ^6^. Understanding how patterns of use vary across tobacco products is therefore important for tobacco control as well as for the prevention and management of nicotine dependence. Monitoring product‐specific trends may help inform public health and addiction‐related policies in response to the rapidly evolving nicotine market^7^.

Japan represents a unique and important setting for studying the diffusion of HTPs, as they were introduced earlier than in many other countries, beginning in the mid‐2010s (around 2015–2017)^8^, ^9^, ^10^, ^11^, ^12^. During the study period, tobacco use patterns were influenced by multiple policy changes, including tobacco tax increases (2018–2021) and the 2020 revision of the Health Promotion Act^13^, which strengthened indoor smoking restrictions while allowing HTP use in designated areas^14^, ^15^, ^16^, ^17^. In addition, Japan's regulatory framework for nicotine products differs from that of many other countries, with HTPs widely available as tobacco products, while nicotine‐containing e‐cigarette liquids are not authorized for sale to general consumers in Japan. HTPs and e‐cigarettes differ in both product design and regulatory status.

Previous studies have made substantial contributions to the understanding of tobacco use patterns in Japan, including the uptake of HTPs. Much of this evidence is derived from internet‐based volunteer sample surveys, such as the Japan “Society and New Tobacco” Internet Survey, which, despite relying on non‐probability samples, have provided detailed insights into trends and behavioral correlates of tobacco use^8^, ^11^, ^12^, ^14^, ^18^, ^19^, ^20^, ^21^, ^22^, ^23^. These studies have documented patterns of HTP uptake, dual use with cigarettes, and associated characteristics, and have highlighted important heterogeneity across demographic groups, such as higher use among male adults. In addition, some studies have attempted to enhance population‐level inference through the application of weighting methods^13^.

However, several important gaps remain. Much of the existing evidence relies on internet‐based samples^13^, ^14^, ^19^, ^24^, which differ methodologically from nationwide probability surveys^25^. Few studies have examined long‐term changes in the composition of tobacco products across multiple survey waves during the rapid diffusion of HTPs^9^, ^11^, ^14^, and nationally representative evidence on changes in the relative prevalence of cigarettes, HTPs, and other products remains limited. It is also unclear whether the expansion of HTP use reflects substitution from combustible cigarettes or concurrent use, particularly in the most recent period, including 2025. Describing overall and product‐specific patterns of tobacco use may therefore provide useful context for understanding shifts in nicotine use and their policy implications.

The primary aim of this study was to describe long‐term trends in overall, product‐specific, and multiple‐product tobacco use in Japan using nationally representative survey data from 2017 to 2025. Tobacco use was defined as past‐30‐day use to capture recent use across different product types. We also examined whether the patterns of use were consistent with the substitution of conventional cigarettes or complementary use alongside them at the population level. We hypothesized that cigarette use would decline over time, but that increases in HTP use would partly offset this reduction, resulting in only a modest decline in overall tobacco use.

## METHODS

### Survey design and target population

This study used data from the Nationwide General Population Survey on Drug Use in Japan, a repeated national survey conducted biennially since 1995 to monitor trends in the use of tobacco, alcohol, pharmaceuticals, and illicit drugs^26^, ^27^, ^28^, ^29^, ^30^. The target population consisted of residents aged 15–64 years, a standard age range consistently applied across survey waves to ensure comparability over time. The survey was designed to be nationally representative. We analyzed data from the 2017, 2019, 2021, 2023, and 2025 survey waves.

The Nationwide General Population Survey on Drug Use in Japan was established in 1995 as a household survey of substance use. Although the survey initially targeted residents aged 15 years or older, the target population was restricted to those aged 15–64 years from the 2009 survey onward^31^. The upper age limit was introduced because the survey focused primarily on substance‐use problems from adolescence through middle adulthood, while population aging was increasing the allocation of sampled participants to older age groups; survey quality and efficiency were also considered^31^. The present study retained this prespecified age range, which was applied consistently across all survey waves from 2017 to 2025. Although the survey was not designed based on the WHO Global Adult Tobacco Survey (GATS), its lower age limit of 15 years is consistent with that used in GATS^32^. Participants aged 15–19 years were included because they formed part of the prespecified target population of the nationwide substance‐use survey.

### Stratified cluster sampling methodology

This survey employed a stratified two‐stage random sampling method to select participants without replacement. Each annual survey was based on an independent cross‐sectional sample drawn using the same stratified two‐stage sampling design. First, Japan was divided into 11 regions (Supporting Table 1). Sampling from the Basic Resident Register was conducted without replacement within each survey year. These regions were further classified into five categories based on the urban scale (metropolitan areas, cities with populations of 200,000 or more, cities with populations of 100,000 or more, cities with populations under 100,000, and rural areas), forming a total of 65 strata. Census tracts were used as the sampling units in the first stage. Within each stratum, participants were selected using probability proportional to size (PPS) sampling. The size measure for PPS was the population aged 15–64 years in each census tract based on the 2016 and 2020 censuses. In the second stage, participants were selected from the Basic Resident Register at each sampling unit using equal‐interval sampling. The basic sample design for the 2017, 2023, and 2025 surveys was to draw approximately 5000 individuals nationwide, whereas the 2019 and 2021 surveys drew approximately 7000 individuals (Supporting Table 2). The number of survey locations was set at 250 for each year, and the sample size was allocated such that the number of individuals selected per location ranged from 13 to 23. For analysis, each survey year was treated as an independent sample; therefore, the stratum for analysis was specified as “Survey Year × Sampling Stratum.” As some sampling locations could be selected from multiple survey waves, primary sampling units (PSUs) were defined as a unique combination of the survey year and sampling location to maintain independence across survey waves.

### Survey implementation procedures

Surveys conducted between 2017 and 2025 were anonymous, using self‐administered questionnaires administered from October to December, based on a consistent sampling framework across survey waves. In 2017 and 2019, responses were collected using paper questionnaires only, whereas from 2021 onward, both paper and online response options were available. Selected participants were mailed a study information sheet, questionnaire, instructions for online responses (from 2021 onward), and a ballpoint pen.

Data collection was performed by a contracted survey organization to ensure standardized procedures. No personal identifiers, such as names, were collected, and participants were assigned anonymized identification numbers. The research team received only anonymized datasets and had no access to IP addresses or other identifying information.

### Ethical considerations

This study was approved by the ethics committees of the authors' institutions (2017, 2019, and 2021 surveys: A2017‐011; 2023 and 2025 surveys: A2023‐031). Participation was voluntary. Participants were provided with an information sheet outlining the study purpose, the voluntary nature of participation, and the handling of results. Consent was indicated by completing the questionnaire, with a confirmation section included at the beginning of both paper and online surveys. The survey was conducted anonymously and in accordance with the Declaration of Helsinki.

### Study population

Responses lacking the information necessary for a complex sampling design (age and sex) were excluded. In addition, responses with less than 50% completion of the questionnaire items were excluded^33^. The proportion of missing data in the primary analysis variables was low (≤1.3%); therefore, multiple imputations were not performed, and complete case analyses were conducted.

### Survey items, outcomes, and covariates

The questionnaire consisted of 53–58 items covering multiple domains, including sociodemographic characteristics and tobacco use. For the present study, only tobacco use items with identical wording across all survey waves (2017–2025) were included to ensure comparability over time. The full questionnaire is available in the original survey reports^26^, ^27^, ^28^, ^29^, ^30^.

The primary outcomes were past‐30‐day indicators of tobacco use, including any tobacco use, product‐specific use, and multiple‐product use. Past‐30‐day use was selected as it is commonly used to represent current use in tobacco surveillance and allows comparisons with previous studies.

Tobacco use was assessed using a multiple‐response question: “Which types of tobacco products have you used during the past 30 days? (Select all that apply.)” Response options included “none,” “cigarettes,” “HTPs,” “e‐cigarettes,” “cigars,” “other tobacco products,” and “unknown type but smoked something during the past 30 days.” Responses indicating “none” together with any other option were considered logically inconsistent and treated as missing. In this study, HTPs referred to devices that heat‐processed tobacco material or tobacco sticks, whereas e‐cigarettes referred to devices that heat a liquid to generate an aerosol without using tobacco leaf. E‐cigarettes were classified irrespective of nicotine content because nicotine content was not separately assessed in all survey waves.

“Any tobacco use” was defined as reporting the use of any of these products during the past 30 days.

For product‐specific analyses, we focused on cigarettes, HTPs, and e‐cigarettes. Binary indicators were created for each product, indicating whether the respondent had used the product at least once in the past 30 days. As the questionnaire allowed multiple responses, each product was analyzed as an independent binary indicator. In the 2025 survey, the response options for e‐cigarettes were further divided into “e‐cigarettes with nicotine” and “e‐cigarettes without nicotine.” To maintain comparability across the survey years, these categories were combined into a single e‐cigarette category and analyzed as a single variable.

Multiple‐product uses are defined based on these three product categories. Respondents reporting the use of two of the three products were classified as dual users, and those reporting the use of all three products were classified as poly‐tobacco users. The use of cigars or other tobacco products was included in the definition of “any tobacco use” but was not included in the multiple‐product use classifications.

To assess internal consistency, an additional question on past‐30‐day smoking was used to identify logically inconsistent responses. These checks were conducted according to predefined data‐cleaning rules and applied consistently across all survey waves before the analysis.

Covariates included survey year (categorical), sex, and age (continuous). In addition, sensitivity analyses were conducted for 2021–2025 by adjusting for response mode (paper vs. online).

### Statistical analyses

This study conducted analyses by considering complex sampling designs based on stratified two‐stage sampling. The strata were defined as 65 strata, combining regional blocks and urban size classifications, with survey locations serving as the PSUs. The same stratum definitions (65 strata) and number of locations (250) were used in each survey year. A representative example of the sample allocation for 2025 is presented in Supporting Table 1. Each survey year was used as an independent sample. Since the same location could be included in multiple years, the analysis specified “survey year × survey location” as a unique PSU.

Individual analysis weights were calculated as inverse probability weights based on the sampling probability at the survey sites and the response probability within sites. The survey weights incorporated both the probability of selection and an adjustment for non‐response within each survey location. The Taylor linearization method was used to estimate the variance. The distribution of analysis weights was examined, and no extreme values requiring trimming were identified; therefore, weight trimming was not applied.

In the primary analyses, annual marginal predicted prevalence estimates were calculated for tobacco use indicators, including tobacco use, individual tobacco product (cigarettes, HTPs, and E‐cigarettes) use, and multiple‐product use categories, defined as cigarettes + HTP, cigarettes + E‐cigarettes, HTP + E‐cigarettes, and cigarettes + HTP + E‐cigarettes. Regression standardization adjusted for age and sex was used to obtain the estimates for each survey year. Specifically, survey‐weighted logistic regression models were fitted for each outcome (past‐30‐day use: yes/no), with survey year entered as a categorical independent variable, and age (continuous) and sex included as covariates.

To improve the comparability of prevalence estimates across survey waves, marginal predicted probabilities were obtained using g‐computation (marginal standardization), with the age and sex distribution of the 2025 survey sample used as the standard population. Marginal predicted probabilities were standardized to the age and sex distribution of the 2025 survey sample. Because the official 2025 national census data were not yet available at the time of analysis, this distribution was used as a proxy for the most recent population structure. This approach provides a consistent and temporally relevant reference for comparing estimates across survey waves. Marginal predicted probabilities were averaged over the weighted study population. Standardized prevalence estimates and 95% confidence intervals (95% CIs) were reported. G‐computation was used to allow model‐based adjustment for covariates and to maintain consistency with the survey‐weighted regression framework. For comparison, unstandardized design‐based prevalence estimates were calculated and are presented as Supporting Information.

The overall effect of the survey year was evaluated using a design‐based Wald test from survey‐weighted logistic regression models, in which survey year was entered as a categorical variable. In addition, linear trends over time were assessed using models in which survey year was treated as an ordinal variable (*p* for trend).

Furthermore, since a combined paper and online response method was introduced in surveys conducted from 2021 onward, sensitivity analyses were performed with the response method added as a covariate to assess the impact of the response method. This sensitivity analysis was limited to the data from 2021 to 2025.

As an additional sensitivity analysis, we repeated the primary analyses after restricting the study population to participants aged 20–64 years, in consideration of the legal minimum age for tobacco use in Japan. Estimates were standardized to the weighted age–sex distribution of participants aged 20–64 years in the 2025 survey sample.

All analyses were performed using the R software (version 4.4.3; R Foundation for Statistical Computing, Vienna, Austria) and the *survey* package (version 4.4‐2).

## RESULTS

**Figure 1:**
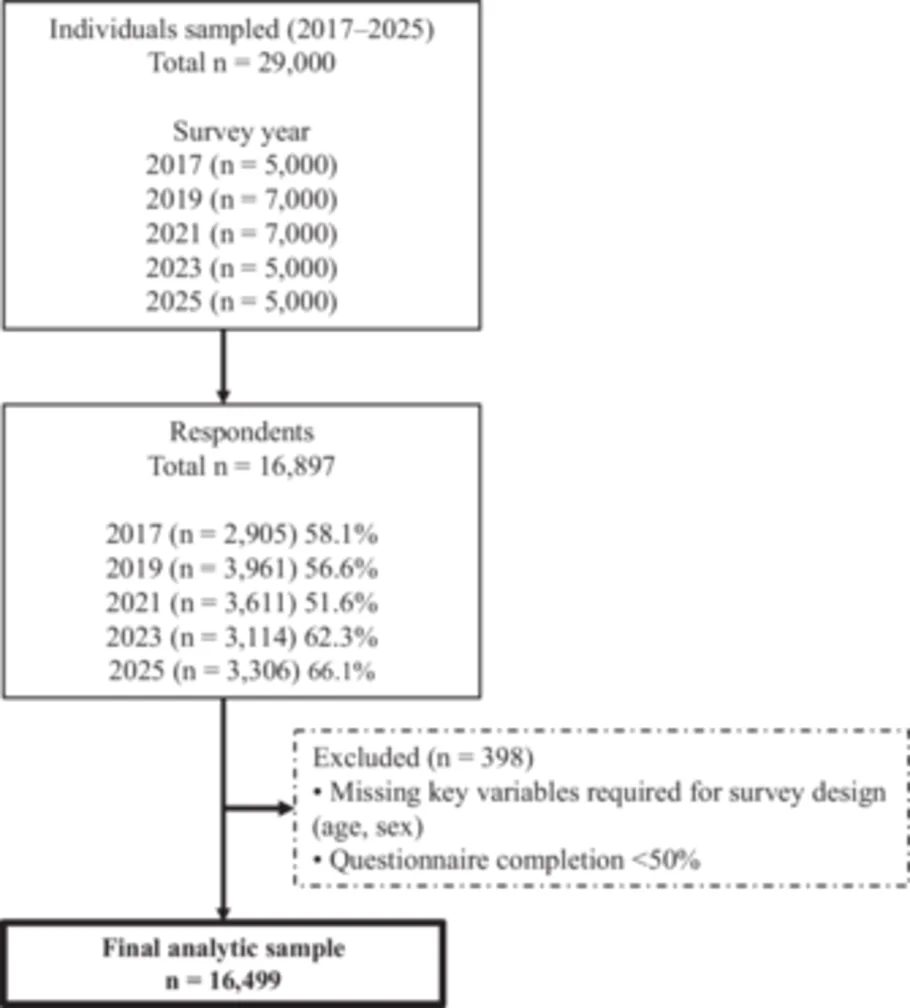
Flow of participants through the study, 2017–2025.

Across the five survey waves, 29,000 individuals were sampled, of whom 16,499 (56.9%) were included in the analytical sample after exclusion (Figure 1). Details of the survey design and weight construction are summarized in Supporting Table 1–4, and participant characteristics are presented in Table 1. Among the analytic sample, 3659 participants reported past‐30‐day tobacco use. Design effects for tobacco use outcomes ranged from 1.06 to 1.42 (Supporting Table 5), indicating modest variance inflation due to the complex sampling design. The precision metrics for the weighted prevalence estimates are presented in Supporting Table 6. The relative standard errors were generally low for the common tobacco use indicators, suggesting acceptable precision. In contrast, several low‐prevalence multiple‐product use indicators showed higher relative standard errors and should, therefore, be interpreted with caution.

**Table 1:** Sample characteristics by survey year (unweighted), 2017–2025.

Variable	Total *n* = 16,499 (%)	2017 *n* = 2899 (%)	2019 *n* = 3942 (%)	2021 *n* = 3476 (%)	2023 *n* = 3026 (%)	2025 *n* = 3156 (%)	Missing rate (%)
**Sex**							
Male	7946 (48.2)	1410 (48.6)	1903 (48.3)	1647 (47.4)	1492 (49.3)	1494 (47.3)	0.0
Female	8553 (51.8)	1489 (51.4)	2039 (51.7)	1829 (52.6)	1534 (50.7)	1662 (52.7)	0.0
Age, mean (SD)	43.5 (13.6)	43.2 (13.4)	43.3 (13.7)	43.8 (13.6)	43.5 (13.7)	43.7 (13.8)	0.0
**Age group (years)**							
15–24	2068 (12.5)	344 (11.9)	504 (12.8)	425 (12.2)	382 (12.6)	413 (13.1)	0.0
25–34	2290 (13.9)	419 (14.5)	542 (13.7)	463 (13.3)	443 (14.6)	423 (13.4)	0.0
35–44	3476 (21.1)	692 (23.9)	863 (21.9)	703 (20.2)	598 (19.8)	620 (19.6)	0.0
45–54	4432 (26.9)	724 (25.0)	1044 (26.5)	996 (28.7)	821 (27.1)	847 (26.8)	0.0
55–64	4233 (25.7)	720 (24.8)	989 (25.1)	889 (25.6)	782 (25.8)	853 (27.0)	0.0
**Past‐30‐day tobacco use**							
Any tobacco use	3,659 (22.3)	715 (24.7)	902 (23.2)	719 (20.8)	632 (21.0)	691 (22.0)	0.8
**Product‐specific use (past 30 days)**							
Cigarette use	2,615 (16.1)	593 (21.0)	690 (17.8)	515 (14.9)	416 (13.8)	401 (12.8)	1.3
HTP use	1448 (8.9)	154 (5.4)	341 (8.8)	277 (8.0)	295 (9.8)	381 (12.2)	1.3
E‐cigarette use	176 (1.1)	30 (1.1)	54 (1.4)	25 (0.7)	21 (0.7)	46 (1.5)	1.3
**Multiple‐product use (past 30 days)**							
Cigarette + HTP	574 (3.5)	72 (2.5)	169 (4.4)	101 (2.9)	98 (3.3)	134 (4.3)	1.3
Cigarette + E‐cigarette	97 (0.6)	17 (0.6)	34 (0.9)	15 (0.4)	7 (0.2)	24 (0.8)	1.3
HTP + E‐cigarette	56 (0.3)	3 (0.1)	20 (0.5)	9 (0.3)	3 (0.1)	21 (0.7)	1.3
Cigarette + HTP + E‐cigarette	41 (0.3)	2 (0.1)	16 (0.4)	8 (0.2)	2 (0.1)	13 (0.4)	1.3
**Response mode**							
Paper	13,499 (81.8)	2899 (100.0)	3942 (100.0)	2007 (57.7)	1685 (55.7)	1497 (47.4)	0.0
Online	3,000 (18.2)	0 (0.0)	0 (0.0)	1469 (42.3)	1341 (44.3)	1659 (52.6)	0.0
**Region**							
Hokkaido	718 (4.4)	147 (5.1)	159 (4.0)	168 (4.8)	112 (3.7)	132 (4.2)	0.0
Tohoku	1,251 (7.6)	218 (7.5)	330 (8.4)	271 (7.8)	226 (7.5)	206 (6.5)	0.0
Kanto	5352 (32.4)	879 (30.3)	1193 (30.3)	1101 (31.7)	1062 (35.1)	1117 (35.4)	0.0
Hokuriku	755 (4.6)	141 (4.9)	204 (5.2)	155 (4.5)	126 (4.2)	129 (4.1)	0.0
Tousan	715 (4.3)	131 (4.5)	201 (5.1)	137 (3.9)	130 (4.3)	116 (3.7)	0.0
Tokai	1,808 (11.0)	325 (11.2)	429 (10.9)	379 (10.9)	340 (11.2)	335 (10.6)	0.0
Kinki	2500 (15.2)	432 (14.9)	589 (14.9)	526 (15.1)	441 (14.6)	512 (16.2)	0.0
Chugoku	972 (5.9)	165 (5.7)	255 (6.5)	205 (5.9)	169 (5.6)	178 (5.6)	0.0
Shikoku	483 (2.9)	94 (3.2)	119 (3.0)	103 (3.0)	88 (2.9)	79 (2.5)	0.0
Northern Kyushu	1128 (6.8)	213 (7.3)	264 (6.7)	260 (7.5)	192 (6.3)	199 (6.3)	0.0
Southern Kyushu	817 (5.0)	154 (5.3)	199 (5.0)	171 (4.9)	140 (4.6)	153 (4.8)	0.0

*Note*: Values represent unweighted counts (*n*) and percentages (%).

Tobacco use (in the past 30 days) included cigarettes, heated tobacco products, e‐cigarettes, cigars, and other tobacco products.

Dual‐ and triple‐product‐use variables were derived from product‐specific indicators.

Abbreviation: HTP, heated tobacco product.

### Trends in past‐30‐day tobacco use

Regression‐standardized prevalence estimates are presented in Table 2, and crude design‐based estimates are shown in Supporting Table 7. Overall past‐30‐day tobacco use showed a modest decline over the study period, decreasing from 24.3% (95% confidence interval [CI]: 22.6–26.0) in 2017 to 21.0% (19.4–22.7) in 2025. Cigarette use has shown a marked decrease over time, falling from 20.7% (19.0–22.3) in 2017 to 12.3% (11.1–13.6) in 2025. In contrast, HTP use increased from 5.3% (4.4–6.1) in 2017 to 11.7% (10.3–13.0) in 2025. E‐cigarette use remained relatively uncommon throughout the study period, ranging from approximately 0.7% to 1.3%. Although a significant overall survey‐year effect was observed (Wald *p* = 0.026), there was no significant linear trend (*p* for trend = 0.930), indicating non‐linear fluctuations over time. Specifically, prevalence increased in 2019, declined in 2021–2023, and rebounded in 2025.

**Table 2:** Regression‐standardized prevalence of tobacco product use by survey year, standardized to the 2025 age–sex distribution.

Tobacco use category	2017% (95% CI)	2019% (95% CI)	2021% (95% CI)	2023% (95% CI)	2025% (95% CI)
**Past‐30‐day tobacco use**					
Any tobacco use	24.3 (22.6–26.0)	22.8 (21.1–24.6)	20.9 (19.4–22.5)	20.3 (18.7–21.9)	21.0 (19.4–22.7)
**Product‐specific use (past 30 days)**					
Cigarette use	20.7 (19.0–22.3)	17.7 (16.2–19.2)	15.2 (13.8–16.6)	13.3 (11.9–14.7)	12.3 (11.1–13.6)
HTP use	5.3 (4.4–6.1)	8.6 (7.6–9.6)	8.2 (7.0–9.5)	9.8 (8.6–10.9)	11.7 (10.3–13.0)
E‐cigarette use	1.1 (0.6–1.5)	1.3 (0.9–1.7)	0.7 (0.4–1.1)	0.7 (0.4–1.0)	1.3 (0.9–1.7)
**Multiple‐product use (past 30 days)**					
Cigarette + HTP	2.5 (1.9–3.1)	4.3 (3.5–5.1)	3.2 (2.4–3.9)	3.4 (2.7–4.1)	4.1 (3.3–4.9)
Cigarette + E‐cigarette	0.5 (0.3–0.8)	0.8 (0.5–1.1)	0.6 (0.2–1.0)	0.2 (0.1–0.4)	0.7 (0.4–1.0)
HTP + E‐cigarette	0.1 (0.0–0.2)	0.5 (0.2–0.7)	0.3 (0.1–0.5)	0.1 (0.0–0.2)	0.6 (0.3–0.9)
Cigarette + HTP + E‐cigarette	0.1 (0.0–0.2)	0.4 (0.2–0.6)	0.3 (0.1–0.5)	0.0 (0.0–0.1)	0.3 (0.1–0.5)

*Note*: Values represent regression‐standardized prevalence estimates (%) with 95% CIs derived from survey‐weighted logistic regression models standardized to the 2025 age–sex distribution. “Any tobacco use” includes cigarettes, heated tobacco products, e‐cigarettes, cigars, and other tobacco products. Multiple‐product use categories represent the use of two or three of the three focal product categories (cigarettes, HTPs, and e‐cigarettes) during the past 30 days. Prevalence estimates are regression‐standardized to the age and sex distribution of the 2025 survey sample. These values represent counterfactual estimates of what prevalence would have been if each survey year had the same age–sex structure as in 2025, and therefore do not correspond to the observed (crude) prevalence in each year (Supporting Table 7).

Abbreviations: CI, confidence interval; HTP, heated tobacco product.

**Figure 2:**
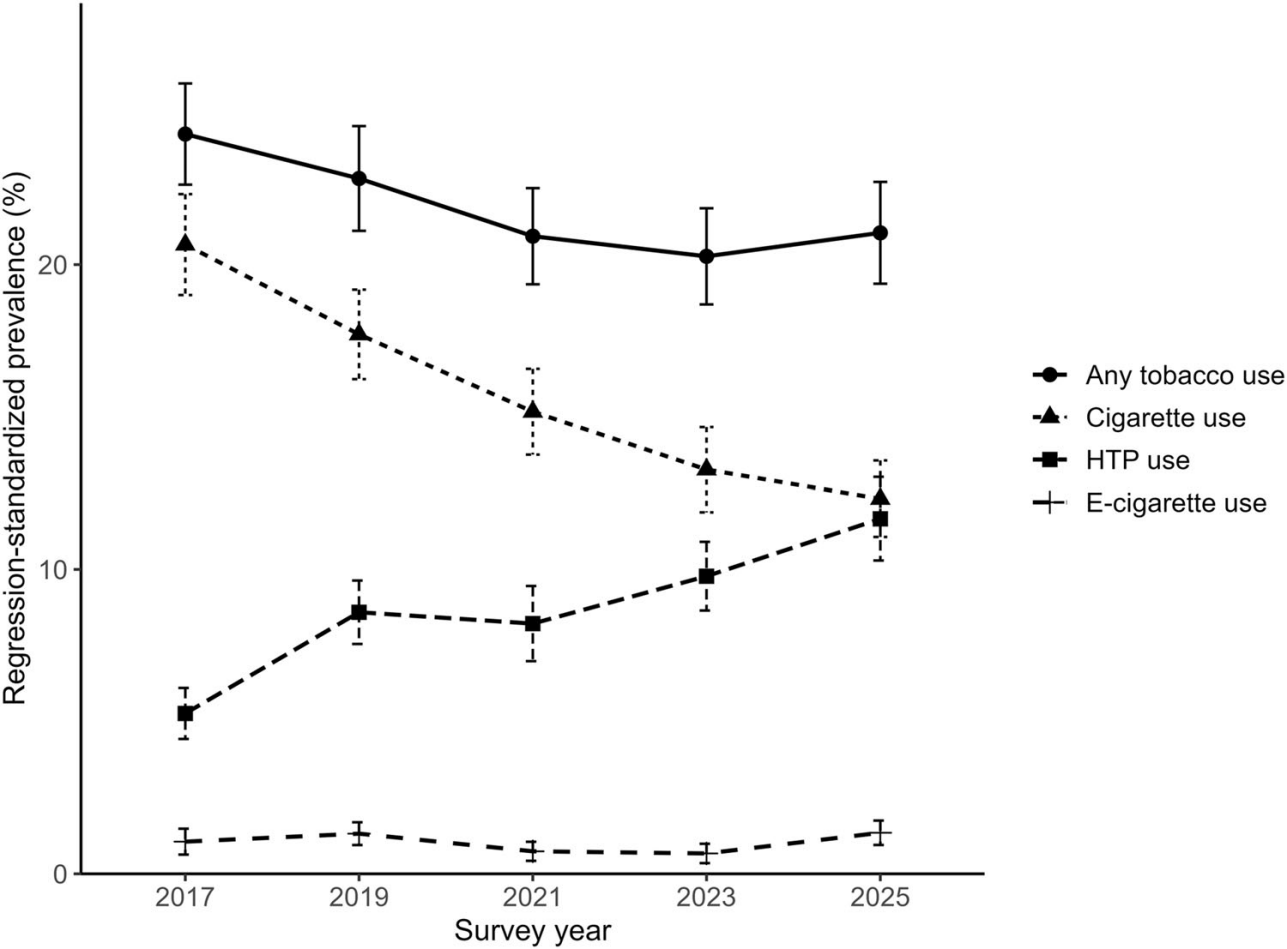
Trends in regression‐standardized prevalence of tobacco product use, Japan, 2017–2025. Points represent regression‐standardized prevalence estimates and error bars represent 95% confidence intervals. Estimates were obtained from survey‐weighted logistic regression models and standardized to the weighted age–sex distribution of the 2025 survey sample. “Any tobacco use” includes cigarettes, heated tobacco products, e‐cigarettes, cigars, and other tobacco products.

**Figure 3:**
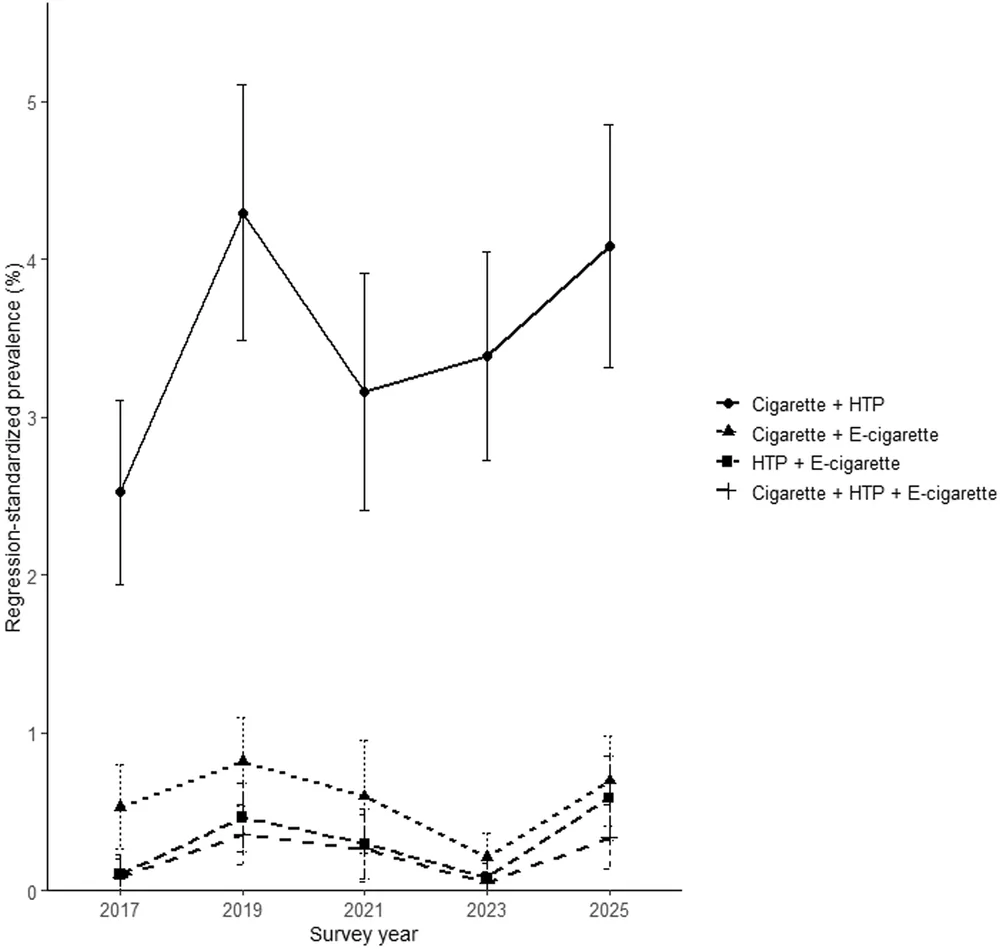
Trends in regression‐standardized prevalence of multiple tobacco product use, Japan, 2017–2025. Points represent regression‐standardized prevalence estimates for dual‐ and triple‐product use and error bars represent 95% confidence intervals. The estimates were standardized to the weighted age–sex distribution of the 2025 survey sample. Multiple‐product use was defined using the following three product categories: cigarettes, heated tobacco products, and e‐cigarettes.

Multicollinearity among covariates (age, sex, and survey year) was assessed, and no evidence of problematic multicollinearity was identified. Cigarette + HTP use increased from 2.5% (1.9–3.1) in 2017 to 4.1% (3.3–4.9) in 2025. Other combinations were rare, including cigarette + E‐cigarette use (0.5% to 0.7%), HTP + E‐cigarette use (0.1% to 0.6%), and triple‐product use (0.1% to 0.3%) (Figures 2 and 3).

Design‐based Wald tests indicated significant survey‐year effects for overall tobacco use and product‐specific use categories (Table 3). Linear trend analyses revealed a significant decreasing trend in cigarette use (20.7% to 12.3%, a reduction of 8.4 percentage points; p for trend <0.001) and a significant increasing trend in HTP use (5.3% to 11.7%, an increase of 6.4 percentage points; *p* for trend <0.001). Overall, tobacco use showed a modest downward trend over the study period (24.3% to 21.0%, a reduction of 3.3 percentage points; *p* for trend <0.001).

**Table 3:** Survey year effects from survey‐weighted logistic regression models for tobacco use outcomes.

Outcome	Overall survey year effect (Wald *p*‐value)	*p* for trend
**Past‐30‐day tobacco use**		
Any tobacco use	0.003	<0.001
**Product‐specific use (past 30 days)**		
Cigarette use	<0.001	<0.001
HTP use	<0.001	<0.001
E‐cigarette use	0.026	0.930
**Multiple‐product use (past 30 days)**		
Cigarette + HTP	0.004	0.044
Cigarette + E‐cigarette	0.026	0.536
HTP + E‐cigarette	0.003	0.063
Cigarette + HTP + E‐cigarette	0.042	0.399

*Note*: Overall survey year effects were evaluated using design‐based Wald tests from survey‐weighted logistic regression models, in which survey year was entered as a categorical variable. For trend analyses, survey year was additionally treated as an ordinal variable and entered as a continuous term, testing for a linear trend over time. Models included age (continuous) and sex as covariates.

Abbreviation: HTP, heated tobacco product.

Among the multiple‐product use categories, cigarette and HTP use showed a weak but statistically significant increasing trend (*p* for trend = 0.044), whereas the other combinations did not exhibit significant linear trends, although some showed overall survey year effects.

### Sensitivity analyses

Sensitivity analyses (2021–2025) showed that HTP use remained robust after adjustment for response mode, whereas the decline in cigarette use was attenuated and no longer significant. E‐cigarette results were consistent, while multiple‐product estimates were less stable (Supporting Tables 8 and 9).

In analyses restricted to participants aged 20–64 years, the product‐specific temporal patterns were consistent with those observed in the primary analysis, including declining cigarette use, increasing HTP use, and persistently low e‐cigarette use. However, the increasing linear trend in concurrent cigarette and HTP use was attenuated and did not reach statistical significance (*p* for trend = 0.071) (Supporting Table 10).

## DISCUSSION

In this nationwide, repeated cross‐sectional survey of the general Japanese population aged 15–64 years, overall tobacco use declined modestly between 2017 and 2025. During this period, cigarette use decreased, whereas HTP use increased and e‐cigarette use remained low. Concurrent use of cigarettes and HTPs was the most common form of multiple‐product use. These findings indicate that the decline in tobacco use occurred alongside a shift in product use patterns rather than a simple reduction. This study adds to the existing literature by providing nationally representative estimates that allow the comparison of temporal patterns during a period of rapid HTP diffusion in Japan^13^, ^14^, ^19^, ^24^, complementing previous studies on product uptake.

Several contextual factors may have contributed to the observed decline in overall tobacco use. Increases in tobacco taxation and prices during the study period are likely to have reduced consumption^15^, ^16^, while broader societal changes during the COVID‐19 pandemic may also have influenced smoking behaviors^13^, ^34^.

The observed decline in cigarette smoking is broadly consistent with findings from previous studies conducted in Japan. Earlier studies have reported rapid increases in HTP use following its introduction to the Japanese market after 2015, accompanied by partial switching from cigarettes and frequent dual use of the two products^8^, ^19^. Similarly, the official sales statistics published by the Tobacco Institute of Japan show a sustained decline in cigarette sales and an increase in HTP sales during the study period^35^, ^36^.

The increase in HTP use may be partly explained by differences in regulatory and social environments across product types. In Japan, HTPs are treated less restrictively than combustible cigarettes in some indoor settings, potentially increasing opportunities for use. Surveys conducted in Japan have also shown that many HTP users perceive these products as causing less harm to others than do combustible cigarettes, including less harm from secondhand exposure^37^, ^38^. Although such perceptions do not demonstrate that secondhand HTP aerosol is harmless or that HTPs pose lower overall health risks, they may be influenced by reduced‐risk marketing and may increase the perceived convenience and social acceptability of these products. These factors may contribute to HTP uptake and potentially offset declines in cigarette smoking.

From an international perspective, the decline in cigarette smoking observed in this study is consistent with trends in many high‐income countries^39^. However, the types of alternative nicotine products differ across settings^7^, ^40^. In many Western countries, electronic cigarettes have expanded as substitutes for conventional cigarettes^41^, ^42^, whereas in Japan, HTPs have become the dominant alternative, and e‐cigarette use remains limited. A 2025 report showed that cigarette (≈12–13%) and HTP use (≈12%) were far more prevalent than e‐cigarette use (≈3%)^30^. Notably, approximately half of e‐cigarette users reported using nicotine‐containing products. In Japan, nicotine‐containing e‐cigarette liquids are not authorized for domestic commercial sale to general consumers, although limited quantities may be imported for personal use. Because the survey did not assess acquisition routes, we could not distinguish personally imported products from those obtained through unauthorized domestic sales. These findings suggest that demand for nicotine‐containing e‐cigarette products persists despite restrictions on their domestic commercial availability. Overall, tobacco use patterns in Japan appear to reflect the interaction between regulatory frameworks and access to nicotine‐containing products.

The Japanese context provides a useful setting to examine how alternative nicotine products shape population‐level tobacco use. Although cigarette smoking declined, the increase in HTP use and the modest reduction in overall tobacco use suggest that nicotine use may be sustained in different forms. These findings indicate that relying on cigarette smoking prevalence alone may underestimate the persistence of nicotine use and highlight the need to consider all nicotine‐containing products when evaluating tobacco control progress.

Another notable finding was the relatively common concurrent use of cigarettes and HTPs. Consistent with previous studies, many HTP users appear to be current or former cigarette smokers, and dual use is common^9^, ^18^. In the present study, although overall cigarette smoking declined, the prevalence of concurrent cigarette and HTP use increased from 2.5% in 2017 to 4.1% in 2025. This pattern suggests that the uptake of HTPs does not simply reflect direct substitution for conventional cigarettes, but may instead involve complementary use. Such patterns may reflect transitional use, situational use, or incomplete switching from cigarettes to HTPs, as reported in prior studies^18^, ^19^. In such cases, HTPs may be added without complete cessation of cigarette smoking, resulting in continued or sustained nicotine exposure. Thus, product diversification may maintain nicotine use in alternative forms even as cigarette smoking declines. From the perspective of sustained nicotine exposure, the use of HTPs may not necessarily lead to meaningful reductions in health risk. This concern is supported by studies showing that biomarkers of potential harm among HTP users do not differ substantially from those of conventional cigarette smokers, despite claims of reduced risk^6^. However, because this study assessed past‐30‐day use, it cannot distinguish between temporary experimentation and sustained transitions. Accordingly, the findings should be interpreted as population‐level changes in product use patterns rather than direct evidence of individual switching behavior.

This study has some limitations. First, the shift from paper‐only surveys (2017–2019) to mixed‐mode surveys (from 2021) may affect comparability across waves. Second, the repeated cross‐sectional design precludes any assessment of individual‐level transitions. Third, tobacco use was self‐reported and may have been under‐reported because of recall or social desirability bias. Such under‐reporting may have been particularly relevant among participants aged 15–19 years, because tobacco use by persons younger than 20 years is prohibited in Japan. Nevertheless, the anonymous, self‐administered format may have reduced social desirability bias compared with interviewer‐administered surveys. Fourth, residual confounding cannot be excluded despite adjustment for key demographic variables. Finally, the overall analytic participation proportion was 56.9%, raising the possibility of non‐response bias. Although the survey weights incorporated response probabilities within each survey location, they may not fully consider differences in tobacco use between respondents and non‐respondents. For example, if people who used tobacco were less likely to participate because of lower health‐related engagement or reluctance to disclose tobacco use, prevalence may have been underestimated. Conversely, the direction of bias cannot be determined because tobacco‐use information was unavailable for non‐respondents.

Tobacco use in Japan shifted between 2017 and 2025, with declining cigarette use, increasing HTP use, persistently low e‐cigarette use, and rising cigarette–HTP dual use. These changes suggest only modest reductions in overall tobacco use and underscore the need for continued surveillance of emerging nicotine products.

## AUTHOR CONTRIBUTIONS

Satomi Mizuno contributed to the study conception and design, data analysis and interpretation, and drafting of the manuscript. Satoshi Inoura, Toshihiko Matsumoto, and Takuya Shimane contributed to the study conception and design, interpretation of the findings, and critical revision of the manuscript. All authors reviewed and approved the final version of the manuscript.

## CONFLICT OF INTEREST STATEMENT

The authors declare no conflicts of interest.

## ETHICS STATEMENT

This study was approved by the ethics committees of the authors' institutions (2017, 2019, and 2021 surveys: A2017‐011; 2023 and 2025 surveys: A2023‐031).

## PATIENT CONSENT STATEMENT

N/A

## CLINICAL TRIAL REGISTRATION

N/A

## Supporting information

Supporting File 1.

## Data Availability

The data that support the findings of this study are available from the corresponding author upon reasonable request and are subject to the completion of the appropriate institutional procedures. The data are not publicly available due to privacy or ethical restrictions.

## References

[1] Simonavicius E , McNeill A , Shahab L , Brose LS . Heat‐not‐burn tobacco products: a systematic literature review. Tob Control. 2019;28:582–594. 10.1136/tobaccocontrol-2018-054419 30181382 PMC6824610

[2] Bialous SA , Glantz SA . Heated tobacco products: another tobacco industry global strategy to slow progress in tobacco control. Tob Control. 2018;27:s111–s117. 10.1136/tobaccocontrol-2018-054340 30209207 PMC6202178

[3] Lempert LK , Glantz SA . Heated tobacco product regulation under US law and the FCTC. Tob Control. 2018;27:s118–s125. 10.1136/tobaccocontrol-2018-054560 30291201 PMC6204223

[4] Lempert LK , Glantz S . Analysis of FDA's IQOS marketing authorisation and its policy impacts. Tob Control. 2021;30:413–421. 10.1136/tobaccocontrol-2019-055585 PMC7952009 32601147

[5] Hammond D , Reid JL . Trends in vaping and nicotine product use among youth in Canada, England and the USA between 2017 and 2022: evidence to inform policy. Tob Control. 2025;34:115–118. 10.1136/tc-2023-058241 37940402 PMC11610498

[6] Glantz SA . PMI's own in vivo clinical data on biomarkers of potential harm in Americans show that IQOS is not detectably different from conventional cigarettes. Tob Control. 2018;27:s9–s12. 10.1136/tobaccocontrol-2018-054413 30131374 PMC6202159

[7] World Health Organization . WHO report on the global tobacco epidemic, 2025: warning about the dangers of tobacco 2025. https://www.who.int/publications/i/item/9789240112063 (accessed March 11, 2026).

[8] Tabuchi T , Shinozaki T , Kunugita N , Nakamura M , Tsuji I . Study profile: The Japan “Society and New Tobacco” Internet Survey (JASTIS): A longitudinal internet cohort study of heat‐not‐burn tobacco products, electronic cigarettes, and conventional tobacco products in Japan. J Epidemiol. 2019;29:444–450. 10.2188/jea.JE20180116 30318495 PMC6776477

[9] Hori A , Tabuchi T , Kunugita N . The spread of heated tobacco product (HTP) use across various subgroups during 2015–16 and 2017–18 in Japan. Environ Health Prev Med. 2023;28:5. 10.1265/ehpm.22-00219 36653145 PMC9884561

[10] Tabuchi T , Kiyohara K , Hoshino T , Bekki K , Inaba Y , Kunugita N . Awareness and use of electronic cigarettes and heat‐not‐burn tobacco products in Japan. Addiction. 2016;111:706–713. 10.1111/add.13231 26566956

[11] Odani S , Tabuchi T . Prevalence and denial of current tobacco product use: Combustible and heated tobacco products, Japan, 2022. Prev Med Rep. 2022;30:102031. 10.1016/j.pmedr.2022.102031 36531095 PMC9747627

[12] Kitajima T , Hisamatsu T , Kanda H , Tabuchi T . Nicotine dependence based on the tobacco dependence screener among heated tobacco products users in Japan, 2022–2023: The JASTIS study. Neuropsychopharmacol Rep. 2025;45(1): e12512. 10.1002/npr2.12512 39780643 PMC11711695

[13] Togawa K , Fong GT , Quah ACK , et al. Impacts of revised smoke‐free regulations under the 2020 Japan Health Promotion Act on cigarette smoking and heated tobacco product use in indoor public places and homes: findings from 2018 to 2021 International Tobacco Control (ITC) Japan Surveys. Tob Control. 2026;35(1):43–51. 10.1136/tc-2024-058697 39419609 PMC12003691

[14] Sutanto E , Miller C , Smith DM , et al. Prevalence, use behaviors, and preferences among users of heated tobacco products: findings from the 2018 ITC Japan Survey. Int J Environ Res Public Health. 2019;16(23): 4630. 10.3390/ijerph16234630 31766410 PMC6926809

[15] Ho LM , Schafferer C , Lee JM , Yeh CY , Hsieh CJ . Raising cigarette excise tax to reduce consumption in low‐and middle‐income countries of the Asia‐Pacific region: A simulation of the anticipated health and taxation revenues impacts. BMC Public Health. 2018;18:1187. 10.1186/s12889-018-6096-z 30340557 PMC6194546

[16] Wan J . Cigarette tax revenues and tobacco control in Japan. Appl Econ. 2006;38:1663–1675. 10.1080/00036840500426900

[17] Ichikawa M , Tabuchi T . Are tobacco prices in Japan appropriate? An old but still relevant question. J Epidemiol. 2022;32:57–59. 10.2188/JEA.JE20210416 34657912 PMC8666320

[18] Hori A , Tabuchi T , Kunugita N . Rapid increase in heated tobacco product (HTP) use from 2015 to 2019: from the Japan “Society and New Tobacco” Internet Survey (JASTIS). Tob Control. 2020;30:474–475. 10.1136/tobaccocontrol-2020-055652 32503900 PMC8237184

[19] Tabuchi T , Gallus S , Shinozaki T , Nakaya T , Kunugita N , Colwell B . Heat‐not‐burn tobacco product use in Japan: its prevalence, predictors and perceived symptoms from exposure to secondhand heat‐not‐burn tobacco aerosol. Tob Control. 2018;27:e25–e33. 10.1136/tobaccocontrol-2017-053947 29248896 PMC6073918

[20] Odani S , Tsuno K , Agaku IT , Tabuchi T . Heated tobacco products do not help smokers quit or prevent relapse: a longitudinal study in Japan. Tob Control. 2024;33:472–480. 10.1136/TC-2022-057613 36849258

[21] Sugiyama T , Tabuchi T . Use of multiple tobacco and tobacco‐like products including heated tobacco and E‐Cigarettes in Japan: a cross‐sectional assessment of the 2017 JASTIS study. Int J Environ Res Public Health. 2020;17:2161. 10.3390/ijerph17062161 32213924 PMC7143444

[22] Fischer K , Bajec M , Mainy N , AlMoosawi S , Sieverding M , Zwisele B , et al. Trends in prevalence and patterns of use of a heated tobacco product (IQOS (™)) in Japan: A three‐year repeated cross‐sectional study. F1000Research. 2022;11:720. 10.12688/f1000research.122491.2 41427060 PMC12716265

[23] Kuwabara Y , Kinjo A , Kim H , Minobe R , Maesato H , Higuchi S , et al. Secondhand smoke exposure and smoking prevalence among adolescents. JAMA Network Open. 2023;6:e2338166. 10.1001/jamanetworkopen.2023.38166 37862017 PMC10589809

[24] Afolalu EF , Langer P , Fischer K , Roulet S , Magnani P . Prevalence and patterns of tobacco and/or nicotine product use in Japan (2017) after the launch of a heated tobacco product (IQOS®): a cross‐sectional study. F1000Research. 2021;10:504. 10.12688/f1000research.52407.1 35528952 PMC9069173

[25] Yeager DS , Krosnick JA , Chang L , Javitz HS , Levendusky MS , Simpser A , et al. Comparing the accuracy of RDD telephone surveys and internet surveys conducted with probability and non‐probability samples. Public Opin Q. 2011;75:709–747. 10.1093/poq/nfr020

[26] Shimane T , Mizuno S , Inoura S . Research on the actual situation of drug abuse and dependence and support for the social reintegration of drug‐dependent persons 2023 Summary report 2023:1–165. https://www.ncnp.go.jp/nimh/yakubutsu/report/pdf/J_NGPS_2023.pdf (accessed July 6, 2024).

[27] Shimane T , Inoura S , Kitamura M , et al. Research on the actual situation of drug abuse and dependence and support for the social reintegration of drug‐dependent persons 2021 Summary report 2022:1–152. https://www.ncnp.go.jp/nimh/yakubutsu/report/pdf/J_NGPS_2021.pdf (accessed September 20, 2023).

[28] Shimane T . Research on the actual situation of drug abuse and dependence and support for the social reintegration of drug‐dependent persons 2019 Summary report 2020:1–104. https://www.ncnp.go.jp/nimh/yakubutsu/report/pdf/J_NGPS_2019.pdf (accessed September 20, 2023).

[29] Shimane T . Research on the actual situation of drug abuse and dependence and support for the social reintegration of drug‐dependent persons 2017 Summary report 2018:1–154. https://www.ncnp.go.jp/nimh/yakubutsu/report/pdf/J_NGPS_2017.pdf (accessed September 20, 2023).

[30] Shimane T , Mizuno S , Inoura S . A national survey to grasp the actual situation of drug abuse and dependence, and research on the abuse of cannabis and on recent drug use trends (23KC2005) Research Report 2025 2026:21–200. https://www.ncnp.go.jp/nimh/yakubutsu/report/pdf/J_NGPS_2025.pdf (accessed April 21, 2026).

[31] Wada K , Shimane T , Tachimori H National general population survey on drug use in Japan, 2009. In: Research report on the current status of drug abuse and dependence and social resources for the prevention of re‐abuse. health and labour sciences research grant, comprehensive research on regulatory science of pharmaceuticals and medical devices. Tokyo, Japan: Ministry of Health, Labour and Welfare; 2010. p. 15–95.

[32] World Health Organization . Global adult tobacco survey. World Health Organization; n.d. https://extranet.who.int/ncdsmicrodata/index.php/collections/GATS/ (accessed July 31, 2026).

[33] American Association for Public Opinion Research (AAPOR) . Standard definitions: final dispositions of case codes and outcome rates for surveys. 9th ed. Oakbrook Terrace, IL: AAPOR; 2016.

[34] Kanai M , Kanai O , Tabuchi T . Impact of the COVID‐19 pandemic on changes in tobacco use behavior: a longitudinal cohort study in Japan. J Epidemiol. 2025;35:255–261. 10.2188/JEA.JE20240180 39581593 PMC12066193

[35] Tobacco Institute of Japan . Annual trends in heated tobacco product sales volume and value. Tobacco Institute of Japan 2026. https://www.tioj.or.jp/data/pdf/260529-heated-sales.pdf (accessed July 31, 2026).

[36] Tobacco Institute of Japan . Annual Trends in Cigarette Sales Volume and Value. Tobacco Institute of Japan 2026. https://www.tioj.or.jp/data/pdf/260529-cigarette-sales.pdf (accessed July 30, 2026).

[37] Yuri K , Higashiyama A , Takemura S , Suzuki H , Bassole Epse Brou MAM , Zhang Y , et al. Do perceptions of the harm of heated tobacco products differ by smoking status? A cross‐sectional analysis of the Japan Society and New Tobacco Internet Survey (JASTIS) 2020 in Japan. BMJ Public Health. 2025;3:e002516. 10.1136/bmjph-2024-002516 41069973 PMC12506111

[38] Xu SS , Meng G , Yan M , Gravely S , Quah ACK , Ouimet J , et al. Reasons for regularly using heated tobacco products among adult current and former Smokers in Japan: finding from 2018 ITC Japan Survey. Int J Environ Res Public Health. 2020;17:8030. 10.3390/ijerph17218030 33142757 PMC7663757

[39] Dai X , Gakidou E , Lopez AD . Evolution of the global smoking epidemic over the past half century: strengthening the evidence base for policy action. Tob Control. 2022;31:129–137. 10.1136/tobaccocontrol-2021-056535 35241576

[40] Organisation for Economic Co‐operation and Development (OECD) . Health at a glance 2025: OECD indicators. OECD 2025. https://www.oecd.org/en/publications/health-at-a-glance-2025_8f9e3f98-en/full-report/smoking-and-vaping_23b355e4.html (accessed March 12, 2026).

[41] Tattan‐Birch H , Brown J , Shahab L , Jackson SE . Trends in use of e‐cigarette device types and heated tobacco products from 2016 to 2020 in England. Sci Rep. 2021;11:13203. 10.1038/s41598-021-92617-x 34168216 PMC8225841

[42] McNeill A , Simonavičius E , Brose LS , Taylor E , East K , Zuikova E , et al. Nicotine vaping in England: an evidence update including health risks and perceptions, September 2022. London: Office for Health Improvement and Disparities; 2022.

