# Supplementary material for: Nationwide trends in cigarette, heated tobacco product, e‐cigarette, and multiple‐product use in Japan 2017–2025
Source: PCN Rep. 2026 Sep 4;5(3):e70409. doi: 10.1002/pcn5.70409 (PMC13543303; doi:10.1002/pcn5.70409)
Supplement: Supplementary file 1 [file PCN5-5-e70409-s001.docx]

**Supporting information**

**Supporting Table 1**. **Distribution of sampled participants and survey locations by region and city-size category (2025 survey).**

| Regions | Metropolis | Cities with a population  ≥ 200,000 | Cities with a population  ≥ 100,000 | Cities with a population  < 100,000 | County (Town/Village) | Total |
| --- | --- | --- | --- | --- | --- | --- |
| Hokkaido | 80 (4) | 21 (1) | 31 (2) | 32 (2) | 31 (2) | 195 (11) |
| Tohoku | 45 (2) | 85 (4) | 32 (2) | 102 (5) | 51 (3) | 315 (16) |
| Kanto | 799 (38) | 451 (21) | 305 (14) | 216 (10) | 68 (3) | 1,839 (86) |
| Hokuriku | 30 (2) | 54 (3) | 22 (1) | 71 (3) | 14 (1) | 191 (10) |
| Tousan | 0 (0) | 39 (2) | 33 (2) | 81 (4) | 30 (2) | 183 (10) |
| Tokai | 154 (9) | 110 (5) | 118 (6) | 105 (5) | 33 (2) | 520 (27) |
| Kinki | 269 (14) | 255 (12) | 99 (5) | 155 (7) | 40 (2) | 818 (40) |
| Chugoku | 76 (5) | 53 (3) | 70 (3) | 51 (3) | 18 (1) | 268 (15) |
| Shikoku | 0 (0) | 58 (3) | 18 (1) | 39 (2) | 19 (1) | 134 (7) |
| Northern Kyushu | 104 (5) | 62 (3) | 39 (2) | 79 (4) | 35 (2) | 319 (16) |
| Southern Kyushu | 29 (2) | 51 (3) | 39 (2) | 63 (3) | 36 (2) | 218 (12) |
| Total | 1586  (81) | 1,239  (60) | 806  (40) | 994  (48) | 375  (21) | 5,000 (250) |

**Note:** Values represent the number of participants sampled in each stratum; numbers in parentheses indicate the number of survey locations (primary sampling units). The sampling strata were defined by a combination of 11 geographic regions and five city-size categories, yielding 65 strata in total. Survey locations were selected within each stratum using probability proportional to size sampling, based on the population size of the census enumeration districts. Approximately 13–23 individuals were sampled from each survey location. The total target sample size for the 2025 survey was 5,000 individuals from 250 locations. The same stratification framework was applied for all survey years (2017–2025).

**Supporting Table 2. Survey design and response characteristics across survey years (2017–2025).**

| **Survey year** | **Target sample size** | **Respondents**  **n (%)** | **Analytic sample**  **n (%)** | **Survey locations**  **(PSUs)** | **Sampling strata** | **Response mode** |
| --- | --- | --- | --- | --- | --- | --- |
| 2017 | 5,000 | 2,905 (58.1) | 2,899 (58.0) | 250 | 65 | Paper |
| 2019 | 7,000 | 3,961 (56.6) | 3,942 (56.3) | 250 | 65 | Paper |
| 2021 | 7,000 | 3,611 (51.6) | 3,476 (49.7) | 250 | 65 | Paper + Online |
| 2023 | 5,000 | 3,114 (62.3) | 3,026 (60.5) | 250 | 65 | Paper + Online |
| 2025 | 5,000 | 3,306 (66.1) | 3,156 (63.1) | 250 | 65 | Paper + Online |

**Note:** The percentages of the respondents and analytical samples were calculated using the target sample size as the denominator. The survey locations were PSUs. The sampling strata were defined by a combination of geographical region and city size (65 strata). All the surveys employed the same stratified two-stage sampling design. PSU, primary sampling unit

**Supporting Table 3. Construction of survey weights: definitions and formulas used in the nationwide resident surveys.**

| **Level** | **Symbol** | **Formula / Value** | **Description** |
| --- | --- | --- | --- |
| Sampling design | — | Stratified two-stage sampling | Sampling design used for the nationwide survey. |
| Stratum | Stratum | Region × city-size category (65 strata) | Sampling strata defined by the combination of 11 geographic regions (Hokkaido, Tohoku, Kanto, Hokuriku, Tousan, Tokai, Kinki, Chugoku, Shikoku, Northern Kyushu, Southern Kyushu) and five city-size categories (metropolis, cities with population ≥200,000, cities with population ≥100,000, cities with population <100,000, counties [towns/villages]). |
| Survey location (PSU) | population | Observed value | Population aged 15–64 in each census enumeration district used as the sampling frame. |
| Survey location (PSU) | Freq | Observed value | Total population aged 15–64 within each stratum used for PPS sampling. |
| Survey location (PSU) | p1 | (population × number of PSUs selected in stratum) / Freq | Probability of selecting each survey location within a stratum under systematic PPS sampling. |
| Individual respondent | sampled | Observed value | Number of individuals sampled within each survey location. |
| Individual respondent | response | Observed value | Number of individuals who responded within each survey location. |
| Individual respondent | p2 | response / sampled | Individual-level response probability within each survey location. |
| Variance estimation method | — | Taylor linearization | Variances were estimated using the Taylor series linearization method to account for the complex survey design. |
| Final weight | weight | 1 / (p1 × p2) | Final survey weight representing the inverse of the combined probability of selection and response. Survey weights were constructed separately for each survey year. |

**Note:** No post-stratification or calibration adjustment was applied. PSU, primary sampling unit; PPS, probability proportional to size

**Supporting Table 4. Distribution of final survey weights: summary statistics.**

| Statistic | Value |
| --- | --- |
| N (non-missing) | 16,499 |
| Mean | 27,105 |
| Standard deviation | 13,868 |
| Minimum | 3,902 |
| 25th percentile | 18,335 |
| Median | 25,000 |
| 75th percentile | 32,945 |
| Maximum | 350,196 |

**Note:** Summary statistics describe the distributions of the final survey weights used in the weighted analyses.

**Supporting Table 5. Design effects, effective sample sizes, and clustering characteristics for tobacco use outcomes.**

| **Category** | **Outcome variable** | **Unweighted N** | **Design effect  (total)** | **Effective sample size** | **Design effect  (weighting)** | **Design effect  (clustering)** | **ICC** | **Mean cluster size** |
| --- | --- | --- | --- | --- | --- | --- | --- | --- |
| Past-30-day tobacco use | Any tobacco use | 16,375 | 1.40 | 11,663 | 1.26 | 1.11 | 0.009 | 13.1 |
| Product-specific use (past 30 days) | Cigarette use | 16,281 | 1.38 | 11,776 | 1.26 | 1.10 | 0.008 | 13.0 |
|  | HTP use | 16,281 | 1.42 | 11,477 | 1.26 | 1.13 | 0.011 | 13.0 |
|  | E-cigarette use | 16,281 | 1.14 | 14,294 | 1.26 | 0.90 | -0.008 | 13.0 |
| Multiple-product use (past 30 days) | Cigarette + HTP | 16,281 | 1.34 | 12,190 | 1.26 | 1.06 | 0.005 | 13.0 |
|  | Cigarette + E-cigarette | 16,281 | 1.17 | 13,866 | 1.26 | 0.93 | -0.006 | 13.0 |
|  | HTP + E-cigarette | 16,281 | 1.06 | 15,318 | 1.26 | 0.84 | -0.013 | 13.0 |
|  | Cigarette + HTP + E-cigarette | 16,281 | 1.11 | 14,690 | 1.26 | 0.88 | -0.010 | 13.0 |

**Abbreviations:** HTP, heated tobacco product; ICC, intraclass correlation coefficient.

**Note:** Design effects and effective sample sizes were estimated using survey-weighted analyses accounting for stratification, clustering, and sampling weights. The total design effect represents the ratio of the variance under a complex survey design to that under simple random sampling. The effective sample size was calculated by dividing the unweighted sample size by the total design effect. The design effects attributable to weighting were approximated using the coefficient of variation of the sampling weights (Kish approximation), and the clustering-related design effects and ICC values were derived from the total design effect. The ICC values were approximate and may have been slightly below zero owing to this approximation.

**Supporting Table 6. Precision of wave-specific prevalence estimates for tobacco outcomes, 2017–2025.**

| **Outcome** | **Survey year** | **Unweighted N** | **Prevalence (%)** | **SE (%)** | **95% CI (%)** | **CI width (pp)** | **RSE (%)** |
| --- | --- | --- | --- | --- | --- | --- | --- |
| **Past-30-day tobacco use** |  |  |  |  |  |  |  |
| Any tobacco use | 2017 | 2890 | 24.4 | 0.9 | 22.7–26.1 | 3.4 | 3.6 |
|  | 2019 | 3883 | 22.9 | 0.9 | 21.2–24.6 | 3.4 | 3.8 |
|  | 2021 | 3453 | 20.9 | 0.8 | 19.2–22.5 | 3.3 | 4.0 |
|  | 2023 | 3008 | 20.7 | 0.8 | 19.0–22.3 | 3.3 | 4.0 |
|  | 2025 | 3141 | 21.1 | 0.9 | 19.4–22.7 | 3.4 | 4.1 |
| **Product-specific use (past 30 days)** |  |  |  |  |  |  |  |
| Cigarette use | 2017 | 2827 | 20.7 | 0.8 | 19.1–22.4 | 3.3 | 4.1 |
|  | 2019 | 3872 | 17.7 | 0.7 | 16.3–19.2 | 2.9 | 4.2 |
|  | 2021 | 3451 | 15.1 | 0.7 | 13.7–16.6 | 2.9 | 4.9 |
|  | 2023 | 3007 | 13.5 | 0.7 | 12.1–15.0 | 2.9 | 5.5 |
|  | 2025 | 3124 | 12.3 | 0.7 | 11.0–13.6 | 2.6 | 5.3 |
| HTP use | 2017 | 2827 | 5.4 | 0.4 | 4.5–6.2 | 1.7 | 8.2 |
|  | 2019 | 3872 | 8.7 | 0.5 | 7.6–9.7 | 2.1 | 6.3 |
|  | 2021 | 3451 | 8.2 | 0.6 | 7.0–9.4 | 2.5 | 7.7 |
|  | 2023 | 3007 | 10.0 | 0.6 | 8.8–11.1 | 2.3 | 5.9 |
|  | 2025 | 3124 | 11.7 | 0.7 | 10.3–13.0 | 2.8 | 6.1 |
| E-cigarette use | 2017 | 2827 | 1.1 | 0.2 | 0.6–1.5 | 0.9 | 20.6 |
|  | 2019 | 3872 | 1.3 | 0.2 | 1.0–1.7 | 0.7 | 14.4 |
|  | 2021 | 3451 | 0.7 | 0.2 | 0.4–1.0 | 0.6 | 21.6 |
|  | 2023 | 3007 | 0.7 | 0.2 | 0.4–1.0 | 0.6 | 24.2 |
|  | 2025 | 3124 | 1.3 | 0.2 | 0.9–1.7 | 0.8 | 15.1 |
| **Multiple-product use (past 30 days)** |  |  |  |  |  |  |  |
| Cigarette + HTP | 2017 | 2827 | 2.6 | 0.3 | 2.0–3.2 | 1.2 | 11.8 |
|  | 2019 | 3872 | 4.4 | 0.4 | 3.5–5.2 | 1.7 | 9.7 |
|  | 2021 | 3451 | 3.2 | 0.4 | 2.4–3.9 | 1.5 | 12.2 |
|  | 2023 | 3007 | 3.5 | 0.4 | 2.8–4.2 | 1.4 | 10.1 |
|  | 2025 | 3124 | 4.1 | 0.4 | 3.3–4.9 | 1.5 | 9.7 |
| Cigarette + E-cigarette | 2017 | 2827 | 0.5 | 0.1 | 0.3–0.8 | 0.5 | 25.9 |
|  | 2019 | 3872 | 0.8 | 0.1 | 0.5–1.1 | 0.6 | 17.8 |
|  | 2021 | 3451 | 0.6 | 0.2 | 0.2–1.0 | 0.7 | 30.8 |
|  | 2023 | 3007 | 0.2 | 0.1 | 0.1–0.4 | 0.3 | 38.0 |
|  | 2025 | 3124 | 0.7 | 0.1 | 0.4–1.0 | 0.6 | 20.8 |
| HTP + E-cigarette | 2017 | 2827 | 0.1 | 0.1 | 0.0–0.2 | 0.3 | 61.1 |
|  | 2019 | 3872 | 0.5 | 0.1 | 0.3–0.7 | 0.4 | 23.6 |
|  | 2021 | 3451 | 0.3 | 0.1 | 0.1–0.5 | 0.4 | 38.0 |
|  | 2023 | 3007 | 0.1 | 0.0 | 0.0–0.2 | 0.2 | 58.3 |
|  | 2025 | 3124 | 0.6 | 0.1 | 0.3–0.9 | 0.5 | 23.0 |
| Cigarette + HTP + E-cigarette | 2017 | 2827 | 0.1 | 0.1 | 0.0–0.2 | 0.2 | 71.8 |
|  | 2019 | 3872 | 0.4 | 0.1 | 0.2–0.6 | 0.4 | 27.6 |
|  | 2021 | 3451 | 0.3 | 0.1 | 0.1–0.5 | 0.4 | 40.9 |
|  | 2023 | 3007 | 0.1 | 0.0 | 0.0–0.1 | 0.1 | 71.1 |
|  | 2025 | 3124 | 0.3 | 0.1 | 0.1–0.5 | 0.4 | 29.8 |

**Abbreviations:** CI, confidence interval; HTP, heated tobacco product; SE, standard error; RSE, relative standard error.

**Notes:** Prevalence estimates are design-based weighted percentages with Taylor-linearized SEs.

RSE was calculated as 100 × SE/prevalence. Estimates with an RSE > 30% were considered statistically unstable and should be interpreted with caution.

**Supporting Table 7. Design-based weighted crude prevalence (%) with 95% confidence intervals for tobacco use, 2017–2025.**

| Outcome | 2017  % (95% CI) | 2019  % (95% CI) | 2021  % (95% CI) | 2023  % (95% CI) | 2025  % (95% CI) |
| --- | --- | --- | --- | --- | --- |
| **Past-30-day tobacco use** |  |  |  |  |  |
| Any tobacco use | 24.4 (22.7–26.1) | 22.9 (21.2–24.6) | 20.9 (19.2–22.5) | 20.7 (19.0–22.3) | 21.1 (19.4–22.7) |
| **Product-specific use (past 30 days)** |  |  |  |  |  |
| Cigarette use | 20.7 (19.1–22.4) | 17.7 (16.3–19.2) | 15.1 (13.7–16.6) | 13.5 (12.1–15.0) | 12.3 (11.0–13.6) |
| HTP use | 5.4 (4.5–6.2) | 8.7 (7.6–9.7) | 8.2 (7.0–9.4) | 10.0 (8.8–11.1) | 11.7 (10.3–13.0) |
| E-cigarette use | 1.1 (0.6–1.5) | 1.3 (1.0–1.7) | 0.7 (0.4–1.0) | 0.7 (0.4–1.0) | 1.3 (0.9–1.7) |
| **Multiple-product use (past 30 days)** |  |  |  |  |  |
| Cigarette + HTP | 2.6 (2.0–3.2) | 4.4 (3.5–5.2) | 3.2 (2.4–3.9) | 3.5 (2.8–4.2) | 4.1 (3.3–4.9) |
| Cigarette + E-cigarette | 0.5 (0.3–0.8) | 0.8 (0.5–1.1) | 0.6 (0.2–1.0) | 0.2 (0.1–0.4) | 0.7 (0.4–1.0) |
| HTP + E-cigarette | 0.1 (0.0–0.2) | 0.5 (0.3–0.7) | 0.3 (0.1–0.5) | 0.1 (0.0–0.2) | 0.6 (0.3–0.9) |
| Cigarette + HTP + E-cigarette | 0.1 (0.0–0.2) | 0.4 (0.2–0.6) | 0.3 (0.1–0.5) | 0.1 (0.0–0.1) | 0.3 (0.1–0.5) |

**Abbreviations:** CI, confidence interval; HTP, heated tobacco product.

**Note:** Estimates represent design-based weighted crude prevalence (%) with 95% CIs accounting for the complex survey design.

**Supporting Table 8. Sensitivity analysis of temporal trends in tobacco use outcomes restricted to the 2021–2025 survey waves with additional adjustment for response mode.**

| **Outcome** | **Overall survey year effect (Wald p-value)** | **P for trend** |
| --- | --- | --- |
| **Past-30-day tobacco use** |  |  |
| Any tobacco use | 0.455 | 0.214 |
| **Product-specific use (past 30 days)** |  |  |
| Cigarette use | 0.833 | 0.668 |
| Heated tobacco product (HTP) use | 0.023 | 0.007 |
| E-cigarette use | 0.016 | 0.016 |
| **Multiple-product use (past 30 days)** |  |  |
| Cigarette + HTP | 0.145 | 0.057 |
| Cigarette + e-cigarette | 0.013 | 0.223 |
| HTP + e-cigarette | 0.003 | 0.037 |
| Cigarette + HTP + e-cigarette | 0.020 | 0.197 |

**Abbreviations:** HTP, heated tobacco product.

**Note:** Sensitivity analyses were conducted by restricting the dataset to the 2021–2025 survey waves and adjusting for response mode (paper vs. web). Survey-weighted logistic regression models were fitted with the survey year as the exposure variable and age, sex, and response mode as covariates. The overall survey year effects were assessed using a design-based Wald test. Linear trends were evaluated by modelling the survey year as an ordinal variable.

**Supporting Table 9. Sensitivity analysis of temporal trends in tobacco use outcomes restricted to the 2021–2025 survey waves without additional adjustment for response mode.**

| **Outcome** | **Overall survey year effect (Wald p-value)** | **P for trend** |
| --- | --- | --- |
| **Past-30-day tobacco use** |  |  |
| Any tobacco use | 0.778 | 0.923 |
| **Product-specific use (past 30 days)** |  |  |
| Cigarette use | 0.011 | 0.003 |
| Heated tobacco product (HTP) use | 0.001 | <0.001 |
| E-cigarette use | 0.014 | 0.021 |
| **Multiple-product use (past 30 days)** |  |  |
| Cigarette + HTP | 0.202 | 0.098 |
| Cigarette + e-cigarette | 0.022 | 0.676 |
| HTP + e-cigarette | 0.004 | 0.124 |
| Cigarette + HTP + e-cigarette | 0.045 | 0.622 |

**Abbreviations:** HTP, heated tobacco product.

**Note:** Sensitivity analyses were conducted by restricting the dataset to the 2021–2025 survey waves. Survey-weighted logistic regression models were fitted with the survey year as the exposure variable and age, sex as covariates. The overall survey year effects were assessed using a design-based Wald test. Linear trends were evaluated by modelling the survey year as an ordinal variable.

**Supporting Table 10. Sensitivity analysis of temporal trends in tobacco use outcomes among participants aged 20–64 years.**

| Outcome | 2017  % (95% CI) | 2019  % (95% CI) | 2021  % (95% CI) | 2023  % (95% CI) | 2025  % (95% CI) | Overall survey year effect (Wald p-value) | P for trend |
| --- | --- | --- | --- | --- | --- | --- | --- |
| **Past-30-day tobacco use** |  |  |  |  |  |  |  |
| Any tobacco use | 25.7  (24.0–27.5) | 24.5  (22.7–26.4) | 22.2  (20.6–23.9) | 21.6  (20.0–23.3) | 22.4  (20.7–24.2) | 0.004 | <0.001 |
| **Product-specific use (past 30 days)** |  |  |  |  |  |  |  |
| Cigarette use | 21.9  (20.2–23.7) | 19.0  (17.4–20.6) | 16.1  (14.6–17.6) | 14.1  (12.6–15.6) | 13.1  (11.8–14.4) | <0.001 | <0.001 |
| HTP use | 5.6  (4.7–6.5) | 9.4  (8.3–10.5) | 8.8  (7.5–10.1) | 10.4  (9.2–11.6) | 12.4  (11.0–13.8) | <0.001 | <0.001 |
| E-cigarette use | 1.1  (0.7–1.6) | 1.4  (1.0–1.8) | 0.8  (0.5–1.1) | 0.7  (0.4–1.1) | 1.4  (1.0–1.8) | 0.051 | 0.807 |
| **Multiple-product use (past 30 days)** |  |  |  |  |  |  |  |
| Cigarette + HTP | 2.7  (2.1–3.3) | 4.7  (3.8–5.6) | 3.4  (2.6–4.2) | 3.5  (2.8–4.2) | 4.3  (3.5–5.1) | 0.002 | 0.071 |
| Cigarette + E-cigarette | 0.6  (0.3–0.9) | 0.9  (0.6–1.2) | 0.6  (0.3–1.0) | 0.2  (0.1–0.4) | 0.7  (0.4–1.0) | 0.033 | 0.419 |
| HTP + E-cigarette | 0.1  (0.0–0.2) | 0.5  (0.3–0.7) | 0.3  (0.1–0.6) | 0.1  (0.0–0.2) | 0.6  (0.3–0.9) | 0.004 | 0.088 |
| Cigarette + HTP + E-cigarette | 0.1  (0.0–0.2) | 0.4  (0.2–0.6) | 0.3  (0.1–0.5) | 0.1  (0.0–0.1) | 0.4  (0.2–0.6) | 0.040 | 0.405 |

**Abbreviations:** CI, confidence interval; HTP, heated tobacco product.

**Note:** Values represent regression-standardized prevalence estimates (%) with 95% confidence intervals derived from survey-weighted logistic regression models adjusted for age and sex and standardized to the weighted age–sex distribution of participants aged 20–64 years in the 2025 survey sample. Overall survey-year effects were evaluated using design-based Wald tests. Linear trends were tested by modeling survey year as an ordinal variable.
